# Bleeding from pancreatic arteriovenous malformation with duodenal ulcer penetration. Case report and literature review

**DOI:** 10.1002/ccr3.2888

**Published:** 2020-05-21

**Authors:** Hiroshi Takayama, Yuichi Shimodate, Satoshi Nomura, Hirohisa Kitagawa, Yoko Akaike, Kaori Uchino, Tetsuo Watanabe, Motowo Mizuno

**Affiliations:** ^1^ Department of Gastroenterology Kurashiki Central Hospital Okayama Japan; ^2^ Department of Surgery Kurashiki Central Hospital Okayama Japan; ^3^ Department of Pathology Kurashiki Central Hospital Okayama Japan; ^4^ Department of Surgery Watanabe Ichoka‐geka Hospital Okayama Japan

**Keywords:** bleeding duodenal ulcer, dynamic computed tomography, pancreatic arteriovenous malformation, penetrated duodenal ulcer, side‐viewing endoscopy

## Abstract

Dilated vessels at the ulcer floor of the second part of the duodenum can be signs of pancreatic arteriovenous malformation; contrast‐enhanced computed tomography should be performed, and surgical treatment should be considered.

## Graphical abstract

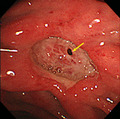

## 1 INTRODUCTION

Pancreatic arteriovenous malformation with duodenal ulcer is a rare condition. Dilated vessels at the ulcer floor of the second part of the duodenum can be signs of pancreatic arteriovenous malformation; contrast‐enhanced computed tomography should be performed to establish the diagnosis, and surgical treatment should be considered.

Pancreatic arteriovenous malformation (P‐AVM) is a rare condition, first reported by Halpern et al in 1968.^1^ It is a vascular anomaly in which blood from the arterial system flows directly into the portal venous system without passing through capillaries in the pancreas. Most patients with P‐AVM are asymptomatic, but some have abdominal pain and/or gastrointestinal bleeding.^2^ A few cases of P‐AVM with abdominal symptoms and duodenal ulcer bleeding have been reported,^3^, ^4^, ^5^, ^6^, ^7^, ^8^, ^9^, ^10^, ^11^ but diagnostic and therapeutic strategies for managing the condition have not been established. In our case, P‐AVM with penetration of a duodenal ulcer was diagnosed with Esophagogastroduodenoscopy (EGD) and dynamic contrast‐enhanced computed tomography (DCE‐CT), and the lesion was treated successfully by operation.

## 2 CASE REPORT

**FIGURE 1:**
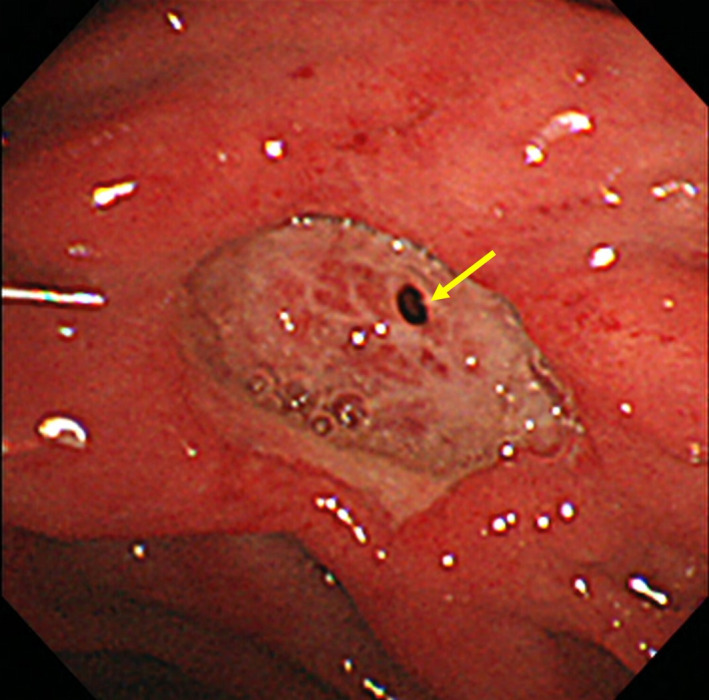
Endoscopic image of perforated duodenal ulcer caused by P‐AVM. Side‐viewing duodenoscopic view of the ulcer in the second part of duodenum; blood vessels and a small hole in the ulcer floor suspicious for micro‐penetration (yellow arrow) are seen

**FIGURE 2:**
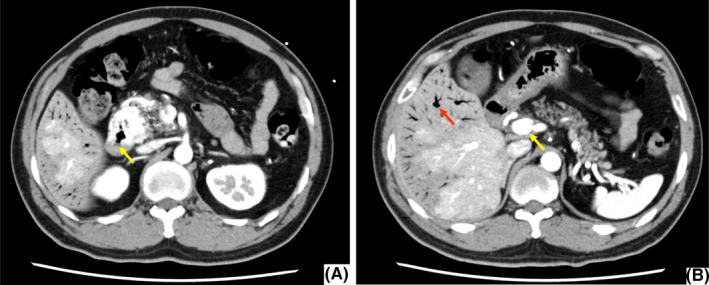
Arterial‐phase images of DCE‐CT. A, A hypervascular lesion in the head of the pancreas extending into the duodenal ulcer (yellow arrow). B, Early enhancement of the portal vein (yellow arrow) and portal venous gas in the liver (red arrows). Swelling of pancreas is not present

A 43‐year‐old man with no relevant medical history had intermittent abdominal pain of two weeks’ duration and hematemesis. He had no fever or signs of peritonitis. The peripheral white blood cell count was 13 000/mm^3^, hemoglobin 16.5 g/dL, and C‐reactive protein 2.25 mg/dL (normal < 0.14 mg/dL). The serum gastrin was 120 pg/mL (normal < 200 pg/mL), and lipase was 13 U/L (normal 13‐55 U/L). Serum tests for cytomegalovirus antigen and *Helicobacter pylori* antibody were negative. EGD revealed a deep ulcer in the second part of duodenum; the ulcer base was not fully seen, but with a side‐viewing duodenoscope, small blood vessels and a small hole at the ulcer floor suggestive of a micro‐penetration of the ulcer were evident (Figure 1). DCE‐CT revealed a hypervascular lesion in the head of the pancreas with extension into the duodenal ulcer and hepatic portal venous gas (Figure 2A, B). The portal vein was enhanced in the arterial phase (Figure 2B). These findings were consistent with the diagnosis of P‐AVM with duodenal ulcer penetration. We performed surgery because his abdominal pain worsened and there was a finding suggestive of penetration.

**FIGURE 3:**
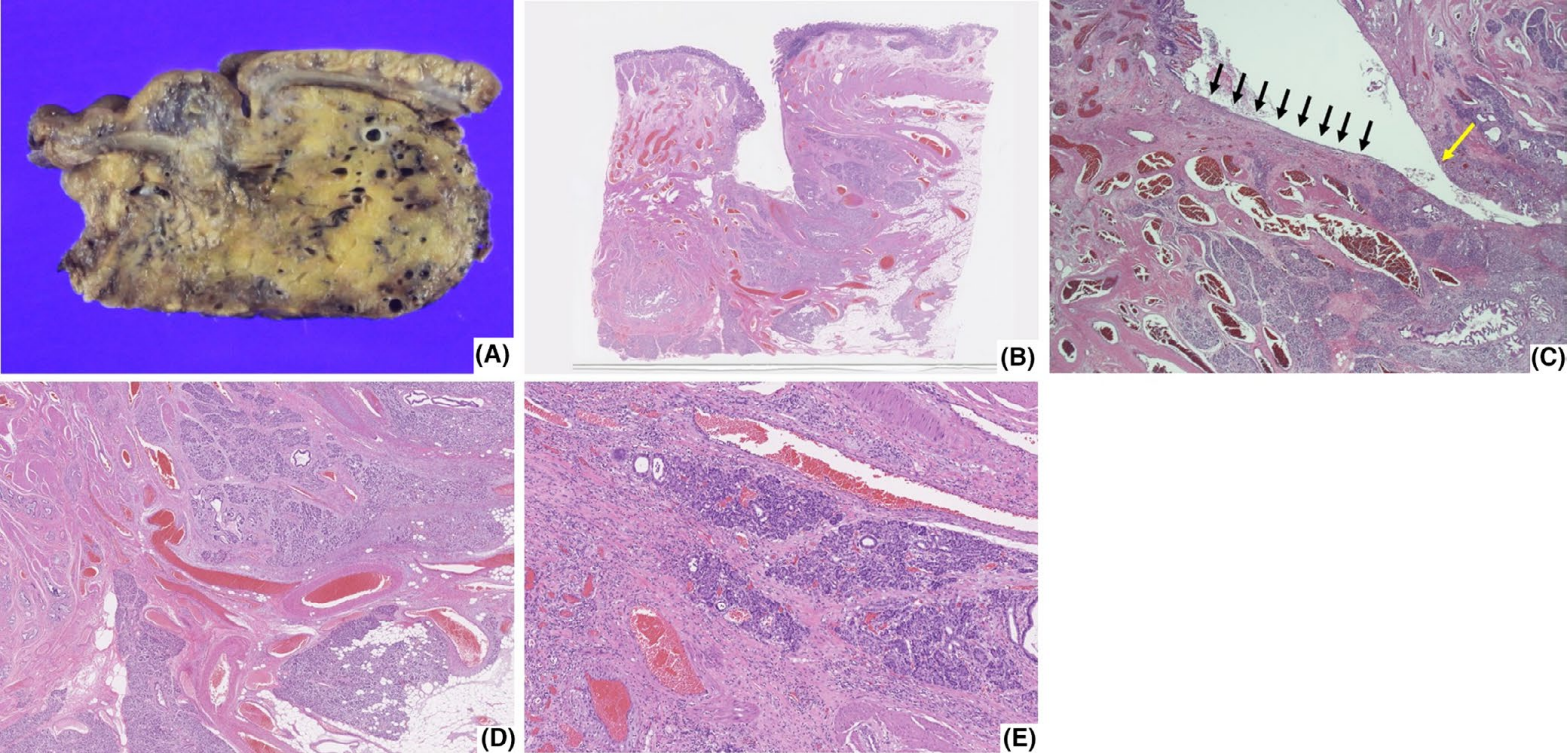
Histopathological findings of the resected specimen. A, A honeycomb‐like structure with dense vessels in the pancreas. B, Abnormal blood vessels are present from the head of the pancreas into the mucosa around the duodenal ulcer (Hematoxylin and Eosin stain, ×0.4). C, There are multiple abnormal blood vessels directly under the ulcer floor (black arrows), and the ulcer perforates to the pancreatic parenchyma (yellow arrow) (Hematoxylin and Eosin stain, ×1.25). D, Conglomeration of irregular dilated and tortuous arteries and veins are present in the pancreatic parenchyma (Hematoxylin and Eosin stain, ×1.25). E, Atrophic pancreatic tissue and infiltration of inflammatory cells are present around the P‐AVM (Hematoxylin and Eosin stain, ×2.5)

The resected specimen had a honeycomb‐like appearance with dense vessels in the pancreas (Figure 3A). Histological evaluation revealed a conglomeration of irregular, dilated and tortuous arteries and veins from the head of the pancreas into the duodenal mucosa through the ulcer (Figure 3B‐D). Atrophic pancreatic tissue and infiltration of inflammatory cells around the P‐AVM also were present (Figure 3E). Duodenal penetration caused by P‐AVM was confirmed. The patient was discharged without postoperative complications 26 days after the operation.

## 3 DISCUSSION

To our knowledge, this is the first description of the endoscopic findings of duodenal ulcer bleeding caused by P‐AVM. Two distinctive characteristics were as follows: (a) ulcer located in the second part of the duodenum and (b) blood vessels of the ulcer floor. Most duodenal ulcers are caused by *H pylori* infection and/or use of nonsteroidal anti‐inflammatory drugs and develop in the duodenal bulb. In contrast, duodenal ulcers associated with P‐AVM usually occur in the second part of the duodenum, as illustrated by the summary of reported cases in Table 1,^3^, ^4^, ^5^, ^6^, ^7^, ^8^, ^9^, ^10^, ^11^ and have no known association with *H pylori* infection. Histologically, multiple abnormal blood vessels are present just under the ulcer floor. The blood vessel‐like findings at the ulcer floor endoscopically visible are most likely abnormal blood vessels of P‐AVM. When such findings are present, P‐AVM should be considered, and DCE‐CT should be performed before endoscopic hemostasis is considered.

**TABLE 1:** Summary of reported cases of P‐AVM with duodenal ulcer

Case	Author	Age	Sex	Ulcer location	Treatment	Reference
1	Inoue S	62	Male	Second portion	Surgery	3
2	Fukami Y	50	Male	Second portion	TAE + Surgery	4
3	Suzuki T	50	Male	Second portion	Surgery	5
4	Regenet N	36	Male	Second portion	Surgery	6
5	Aida K	54	Male	Second portion	Surgery	7
6	Uda O	48	Male	Bulb	TAE + Surgery	8
7	Tano S	52	Male	Second portion	Conservative treatment	9
8	Katoh H	33	Male	Second portion	Surgery	10
9	Arora A	37	Male	Second portion	TAE + Surgery	11

Abbreviation: TAE, transcatheter arterial embolization.

This report conveys important messages concerning management of duodenal ulcer bleeding. Despite advances made in the management of bleeding ulcers, hemostasis is still difficult in certain cases. Actively bleeding, large ulcers (≥2 cm), and exposed vessels (≥2 mm diameter) are factors that predispose to unsuccessful endoscopic hemostasis.^12^, ^13^ Identification of controllable bleeding through endoscopic findings is important to help prevent injudicious attempts at endoscopic hemostasis.

Bleeding from P‐AVM is one of the causes of bleeding duodenal lesions that is difficult to manage endoscopically. Transcatheter arterial embolization and external radiation have been attempted for P‐AVM, but surgical resection is required in most cases.^2^ Indeed, 8 of 9 (89%) of reported patients of P‐AVM with duodenal ulcers were ultimately treated with surgical resection^3^, ^4^, ^5^, ^6^, ^7^, ^8^, ^9^, ^10^, ^11^ (Table 1). We feel that surgical therapy should be the primary treatment for duodenal ulcer bleeding from P‐AVM because there is a possibility of penetration.

Ulcer associated with P‐AVM is believed caused by local ischemia of the mucosa.^9^ In the present case, abnormal blood vessels of P‐AVM were present from the head of the pancreas into the duodenal mucosa at the ulcer site, a finding suggesting that direct compression of P‐AVM caused mucosal ischemia, leading to ulcer formation. It is reported that P‐AVM can cause acute pancreatitis,^7^, ^14^ thus another proposed mechanism for the development of ulcers with P‐AVM is acute pancreatitis secondary to the P‐AVM. In our case, focal pancreatitis around P‐AVM was present histologically, but increased serum pancreatic enzyme values and swelling of pancreas were not present; thus, ulcer penetration may have caused the focal pancreatitis.

In conclusion, we have reported a case of bleeding from P‐AVM with duodenal ulcer penetration. Bleeding from duodenal ulcer in the second part of the duodenum, with blood vessels at the ulcer floor, should prompt suspicion of a P‐AVM and performance of DCE‐CT rather than endoscopic treatment of the ulcer. Surgical treatment of the P‐AVM and ulcer is the preferred management.

## CONFLICT OF INTEREST

The authors declare no conflict of interest regarding this study.

## AUTHOR CONTRIBUTIONS

HT: was involved in the management of the case and wrote the manuscript. YS: was involved in revising of the manuscript. SN: was involved in the surgery. HK: was involved in the surgery. YA: was involved in the pathological diagnosis. KU: was involved in the pathological diagnosis. TW: was involved in the diagnosis with esophagogastroduodenoscopy. MM: was involved in revising the manuscript and final approval.
